# Inflammaging-Driven Osteoporosis: Is a Galectin-Targeted Approach Needed?

**DOI:** 10.3390/ijms26136473

**Published:** 2025-07-04

**Authors:** Marina Russo, Caterina Claudia Lepre, Annalisa Itro, Gabriele Martin, Gianluca Conza, Maria Consiglia Trotta, Monica Puticiu, Anca Hermenean, Francesca Gimigliano, Michele D’Amico, Giuseppe Toro

**Affiliations:** 1 PhD Course in National Interest in Public Administration and Innovation for Disability and Social Inclusion, Department of Mental, Physical Health and Preventive Medicine, University of Campania “Luigi Vanvitelli”, 80138 Naples, Italy; marina.russo@unicampania.it; 2 School of Pharmacology and Clinical Toxicology, University of Campania “Luigi Vanvitelli”, 80138 Naples, Italy; 3 PhD Course in Translational Medicine, University of Campania “Luigi Vanvitelli”, 80138 Naples, Italy; caterinaclaudia.lepre@unicampania.it; 4 Department of Experimental Medicine, University of Campania “Luigi Vanvitelli”, 80138 Naples, Italy; michele.damico@unicampania.it; 5 Multidisciplinary Department of Medical, Surgical and Dental Sciences, University of Campania “Luigi Vanvitelli”, 80138 Naples, Italy; annalisa.itro@studenti.unicampania.it (A.I.); gabriele.martin@studenti.unicampania.it (G.M.); gianluca.conza@studenti.unicampania.it (G.C.); francesca.gimigliano@unicampania.it (F.G.); giuseppe.toro@unicampania.it (G.T.); 6 Faculty of Medicine, Vasile Goldis Western University of Arad, 310144 Arad, Romania; puticiu.monica@uvvg.ro (M.P.); hermenean.anca@uvvg.ro (A.H.); 7 “Aurel Ardelean” Institute of Life Sciences, Vasile Goldis Western University of Arad, 310144 Arad, Romania

**Keywords:** osteoporosis, osteoporotic fractures, Galectins (Gals), Galectin-1 (Gal-1), Galectin-3 (Gal-3)

## Abstract

Osteoporosis (OP) is a chronic disease characterized by reduced bone mass and altered microarchitecture, leading to bone fragility and fractures. Due to its high morbidity, disability, and healthcare costs, identifying new biomarkers and therapeutic strategies is crucial for improving OP diagnosis and prevention. In this context, this narrative review aims to depict the role of carbohydrate-binding proteins Galectins (Gals) in the combined processes of inflammation and aging contributing to bone fragility by exploring their potential as novel therapeutic targets for OP.

## 1. Introduction

**Figure 1:**
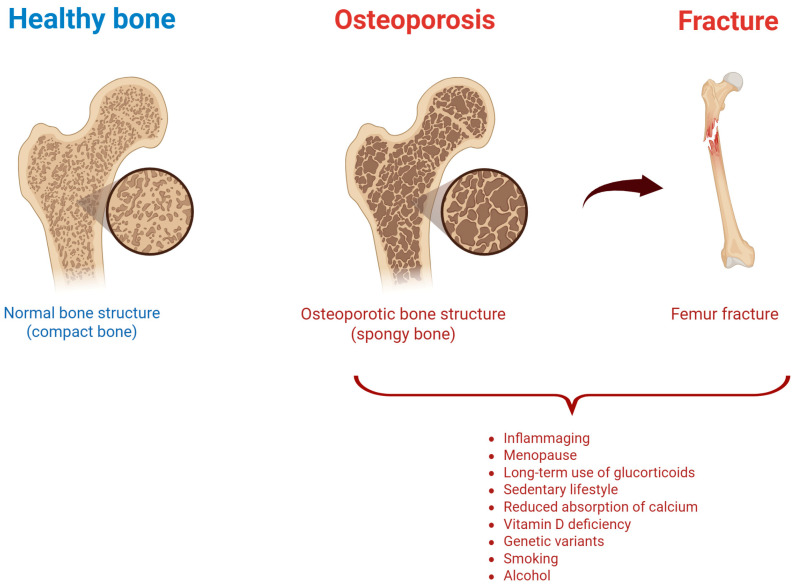
Healthy vs. osteoporotic bone, and risk factors associated with osteoporotic fracture. Created by Biorender.

Osteoporosis (OP) is a chronic musculoskeletal disease characterized by a reduced bone mass and altered microarchitecture, leading to bone fragility [1,2,3] (Figure 1).

OP is the result of an unbalanced bone metabolism, with a bone resorption by osteoclasts higher than bone formation by osteoblasts. Its etiology can be considered multifactorial since hormonal, nutritional, and lifestyle factors play a crucial role in OP onset and progression, as well as genetic modifications [4]. Variants in genes encoding collagen type I alpha 1 (COL1A1), vitamin D receptor, and estrogen receptor have been reported in osteoporotic population [5]. Additionally, some conditions (hyperthyroidism, hyperparathyroidism, and Cushing’s syndrome) could lead to secondary forms of OP [6]. Similarly, diabetes or some autoimmune rheumatologic diseases, such as rheumatoid arthritis, ankylosing spondylitis, and systemic lupus erythematosus, may further increase the OP risk [7,8]. Nutritional deficiencies also play a pivotal role in OP condition: insufficient calcium and vitamin D intake negatively affect bone mineralization [9], and protein deficiency and an excessive intake of phosphorus and sodium can contribute to OP development [10]. Other well-documented risk factors for OP development are physical inactivity (negatively associated with bone density), smoking (contributing to bone oxidative stress), and excessive alcohol consumption (disrupting hormone balance influencing bone homeostasis) [11]. Furthermore, some medications including long-term use of glucocorticoids, anticonvulsants, and certain antiretroviral drugs contribute to bone demineralization and increase the risk of osteoporotic fractures (OF) [12]. These latter are associated with high morbidity and relevant physical disability, leading to reduced quality of life, and high healthcare costs [13,14]. Worthy of note, OF incidence is expected to constantly increase due to the aging of the general population [15,16,17,18]. Overall, despite the high incidence and costs associated with OP, to date a significant percentage of cases remain undiagnosed, only a quarter of diagnosed cases are treated, and an improved drug adherence is still needed [4,19,20].

### The Influence of Inflammaging in OP

After the age of 30, bone mass appears to slowly and progressively decline [21], with a net loss of bone mass and a decreased bone regeneration, due to fewer active osteoblasts combined with a stable or increased osteoclast activity [22,23,24]. Of course, with the increasing age, complex changes negatively affect bone health, leading to an increased OP and OF risk [25]. Indeed, the population over the age of 65 years must undergo in-depth clinical investigations to avoid the onset of fractures [4,26].

**Figure 2:**
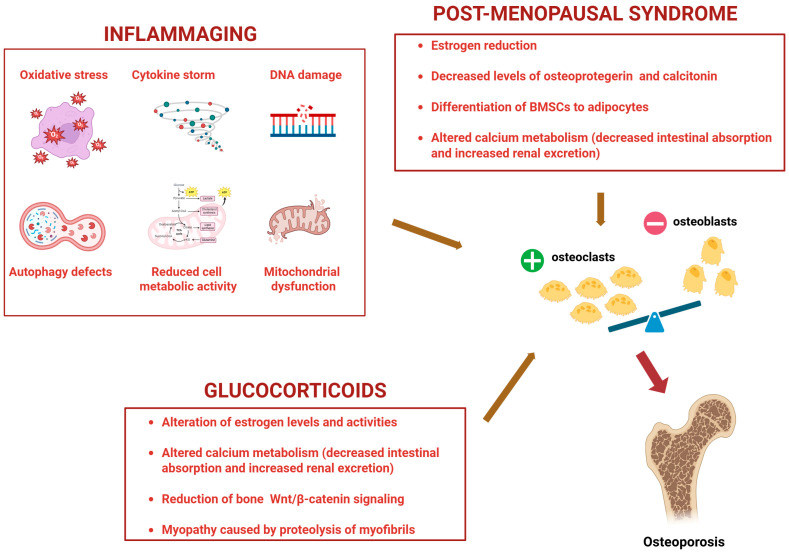
Molecular mechanisms underlying Osteoporosis driven by inflammaging, post-menopausal syndrome, and use of glucocorticoids. BMSCs: Bone marrow mesenchymal stem cells. +: increased; −: decreased. Created by Biorender.

From a molecular point of view, aging leads to a state of chronic low-grade inflammation and cellular senescence, also known as “inflammaging”, which is associated with OP [27,28]. This is underlined by biological and molecular mechanisms different from the ones characterizing post-menopausal syndrome or glucocorticoid use-driven OP (Figure 2).

Processes such as oxidative stress, cytokine storms, DNA damage, autophagy defects, nutrient deprivation/reduced metabolic cell activity, and mitochondrial dysfunction lead to bone inflammaging [29], underlined by the following molecular mechanisms: cell cycle arrest through the upregulation of p16, p21, p53 (markers of DNA damage response); impaired autophagy by the activation of mTOR (an autophagy inhibitor); promotion of senescence by decreased levels of Sirtuins (able to counteract osteoblast senescence); increased type 1 interferon and STING pathway, linking DNA damage, inflammation, and cellular senescence [30].

Aging can also modify hormonal balance, thus contributing to OP [31]. In this regard, the reduction in estrogen in women after menopause and in testosterone in older men affects the activity of osteoclasts and osteoblasts by leading to bone alterations [32,33]. Conversely, the increased levels in the elderly population of parathyroid hormone (PTH), produced by the parathyroid glands, stimulates the activity of osteoclasts, leading to bone weakness [34]. It is well known that aging leads to a reduction in the intestinal absorption of calcium and vitamin D [35], a decreased efficiency in producing vitamin D through sun exposure, and a lower ability of the kidney to convert vitamin D to its active form [36]. Consequently, the body tends to mobilize calcium from the bones to maintain normal blood levels, contributing to bone loss [37]. Another factor contributing to age-related OP development is the progressive loss of muscle mass, known as sarcopenia. Indeed, the muscles play a fundamental role in supporting bones and maintaining their balance and strength, with a bone–muscle molecular crosstalk having a relevant role in determining bone quality [38]. Therefore, the risk of bone fractures increases with muscle mass loss [39].

Overall, the combination of imbalances in bone remodeling, hormonal changes, reduced nutrient absorption, and lifestyle factors in elderly people contributes to progressive bone loss, which can be modified both by lifestyle changes (proper diet, regular physical activity) and pharmacological options.

## 2. OP Treatments: Approved and Ongoing

Regarding the pharmacological options used in the prevention and management of OP and OF, these include antiresorptive drugs, anabolic agents (AAS), and dual-action agents [40,41].

Bisphosphonates are antiresorptive drugs which reduce bone resorption mainly inducing osteoclast apoptosis, and currently represent the standard pharmacological OP therapy [42]. All approved bisphosphonates have been shown to reduce the risk of vertebral fractures and increase bone mineral density [43], with their antiresorptive effect persisting even after their discontinuation [43,44]. Bisphosphonates are taken by oral or intravenous administration. Particularly, alendronate, risendronate, and ibandronate are daily, weekly, or monthly administered, while ibandronate and zoledronate are intravenously given, respectively, every 3 months and once a year [45]. These drugs are characterized by limited toxicity, with the main side effects concerning the upper gastrointestinal tract for oral bisphosphonates, and flu-like symptoms (fever, muscle, and bone) after the first infusion for intravenous bisphosphonates, treated with symptomatic drugs [46,47]. However, bisphosphonates cannot be administered in patients with serious kidney problems [48]. In rare cases and when used for a long time, they have been associated with osteonecrosis of the jaw (caused primarily by avascular necrosis and complicated by secondary infection) and atypical femur fractures [49,50]. Another antiresorptive drug commonly used for the treatment of OP is Denosumab, a monoclonal antibody that binds strongly and specifically to the so-called RANK ligand (RANKL), a key mediator of bone resorption [51,52]. Particularly, Denosumab blocks the interaction between RANKL and RANK on osteoclasts, thus inhibiting their differentiation, activation, and survival [53].

Regarding the AAS, such as the parathyroid hormone analogues teriparatide and abaloparatide, they are used in the OP treatment to stimulate bone formation [54]. Indeed, daily AAS administrations promote osteoblast activity and are generally used in patients with severe OP or not responder to other treatments [55].

More recently, the dual action agent Romosozumab was introduced as a drug able to both stimulate bone formation and reduce bone resorption by acting on the Wnt pathways [56]. This monoclonal antibody, administered as a monthly subcutaneous dose, acts by binding and inhibiting sclerostin (an important regulator of bone formation), leading to a significantly reduction in both vertebral and non-vertebral fractures in recent clinical studies [57,58,59,60,61].

Another pharmacological option in OP management is hormone replacement therapy (HRT). Acting on the hormone imbalance, HRT may consist of taking estrogen alone or in combination with other sex hormones (progestins). This treatment slows bone turnover and increases bone mineral density in all skeletal areas in postmenopausal women of any age [62]. Other OP drugs are selective estrogen receptor modulators (SERMs), binding to estrogen receptors and acting as agonists or antagonists, depending on the organ [63]. Although not a first-line OP treatment, SERMs (tamoxifene, bazedoxifene, and raloxifene) are used in postmenopausal women with both OP and increased risk of breast cancer, decreasing bone resorption and the risk of vertebral fractures [64,65].

Besides the OP drugs currently available, calcium and vitamin D supplementation are certainly useful to reduce OF [66] and should be always recommended [67,68]. Similarly, bone loss and OF-related pain are reduced by calcitonin supply, able to maintain calcium homeostasis, and counterbalance the aged-induced PTH increase [66,69].

Some other therapeutic options are currently under clinical investigation. A randomized control clinical trial evidenced the efficacy of Xulin Jiangu granules, a traditional Chinese medicine, in postmenopausal OP aggravated by renal failure and blood stasis syndrome (NCT03563235). Moreover, the effects of 24-month application of nitroglycerin ointment have been investigated in OP in elderly women (NCT00252421). Great interest has also been recently generated by mesenchymal stem cells (MSCs), since they can be useful in halting OP deterioration by restoring bone turnover, re-establishing the balance between bone formation and resorption, increasing bone mineral density, and reducing bone inflammation [70]. Particularly, a clinical study is testing the intravenous infusion of autologous fucosylated bone MSCs in patients with OP (NCT02566655), while a phase 2 clinical trial, still recruiting patients, aims to evaluate the effectiveness of the allogeneic mesenchymal cell from umbilical cord implanted in OP patients (NCT04501354). Furthermore, the application of a local osteo-enhancement procedure in the proximal femur of OP women is under investigation in a clinical study currently in the recruitment phase (NCT05202678).

From a preclinical point of view, the extracts of Epimedium (Berberidaceae), also known as horny goat weed or Yin Yan Huo, demonstrated several protective effects on musculoskeletal system as a putative modulator of estrogen signaling, RANKL/RANK or reactive oxygen species (ROS) pathways [71,72,73]. In particular, the Epimedium extracts epimedin B and epimedin C significantly stimulated the proliferation of osteoblast-like cells (UMR106) [72,74]. An ovariectomized rat model with OP was treated with icariin, extracted from Epimedium, which inhibited bone loss [75]. Epimedium flavonoids were also used to treat rats in an in vivo model of retinoic acid-induced OP, leading to increased bone mass and improved biomechanical properties of the bone [76].

While bisphosphonates and denosumab are used for OP caused by post-menopausal syndrome, use of glucocorticoids or secondary OP, the strategies aimed to specifically reduce inflammaging-driven OP and improve its outcome are still under investigation. Indeed, only a single recent study associated icariin with an anti-inflammaging effect through the modulation of autophagy in an animal OP model [77].

## 3. Galectin Family and Inflammaging

**Figure 3:**
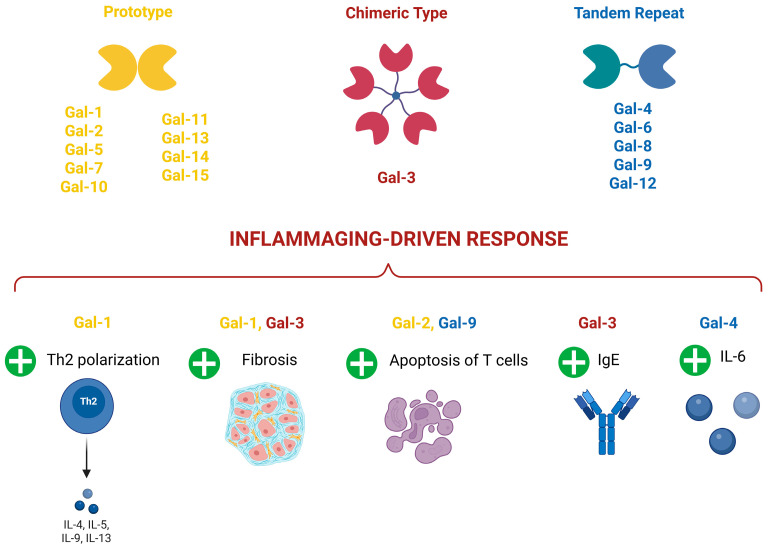
Galectin types and their involvement in inflammaging-driven response. Gal-1: Galectin 1; Gal-2: Galectin 2; Gal-3: Galectin 3; Gal-4: Galectin 4; Gal-5: Galectin-5; Gal-6: Galectin 6; Gal-7: Galectin 7; Gal-8: Galectin 8; Gal-9: Galectin-9; Gal-10: Galectin 10; Gal-11: Galectin 11; Gal-12: Galectin 12; Gal-13: Galectin 13; Gal-14: Galectin 14; Gal-15: Galectin 15; Th2: T helper 2 lymphocytes; IgE: Immunoglobulin E; IL-4: Interleukin 4; IL-5: Interleukin 5; IL-6: Interleukin 6; IL-9: Interleukin 9; IL-13: Interleukin 13. +: increased. Created by Biorender.

Galectins (Gals) are a family of carbohydrate-binding proteins that play a crucial role in the regulation of various cellular processes including aging, inflammation, immune modulation, and tissue remodeling [78]. The family includes 15 members (Figure 3) with a broad tissue distribution, located mainly in the cell cytoplasm, but also translocated into the nucleus [79].

Gal functions can vary depending on the cell-type involved, mainly immune and inflammatory [80]. Overall, Gals are able to regulate cell-cell interaction, apoptosis, epithelial mesenchymal transition, tumor progression, and, particularly, inflammation, aging, and age-related diseases [80].

Particularly, during acute inflammation, Galectin 1 (Gal-1) seems to exert an anti-inflammatory role by controlling neutrophil trafficking and extravasation [81], while Galectin 3 (Gal-3) is characterized by pro-inflammatory actions through the increase in macrophage and neutrophil numbers and phagocytosis [82,83].

In the wide area of inflammaging-related chronic disorders, Gal-1 seems to play a controversial role. Indeed, it has been proposed as an attractive immunosuppressive agent, able to reduce T helper 1 (Th1) and 17 Th17 pro-inflammatory responses and shift the cytokine balance toward a T helper 2 (Th2)-dependent anti-inflammatory polarized profile in experimental models of experimental autoimmune encephalomyelitis (EAE) [84], inflammatory bowel disease [85], Graft versus host disease [86], and experimental autoimmune uveitis [87]. On the contrary, a pro-fibrotic role has been associated with Gal-1 increase both in cardiac and liver fibrosis induced by diabetes [88,89].

Galectin 2 (Gal-2) could exert a protective role during allergic inflammation by inducing apoptosis of CD8+ T cells [90], while Gal-3 seems to exacerbate asthma, atopic dermatitis, and EAE by promoting an (Immunoglobulin E) IgE increase [91,92,93]. Moreover, the increase in Gal-3 and Gal-1 in bronchoalveolar lavage from Coronavirus disease 2019 (COVID-19)-infected patients was correlated with pro-inflammatory mediators favoring lung fibrosis [94].

Galectin 4 (Gal-4) is associated with an increase in the pro-inflammatory interleukin 6 (IL-6) during colitis [95], while Galectin 9 (Gal-9) is associated with an increase in the anti-inflammatory role during EAE by favoring the apoptosis of Th1 cells [96].

Considering more specifically the aging mechanisms, Gal-3 has been recently identified as a receptor for advanced glycosylation end-products (AGEs) [97]. Accordingly, serum Gal-3 concentration significantly correlated with frailty in elder people [98], with lower Gal-3 levels associated with successful ageing [99]. An increase in Gal-3 has also been correlated with neuroinflammation and neurodegeneration in an experimental model of aged-induced neurodegeneration [100]. Conversely, Gal-3 genetic deletion seems to exacerbate age-related myocardial hypertrophy and fibrosis in mice [101]. In Alzheimer’s disease (AD), the number of microglial cells expressing Gal-1 and Gal-3 tends to increase with age [102,103]. Moreover, microglia-derived Gal-9 seems to favor amyloid-β accumulation in experimental AD [104] (Figure 3).

### 3.1. Gal-1 and Gal-3 in Bone Fragility, Resorption, and Senescence

Except for a single study reporting an association between Galectin 8 (Gal-8) with hyperactive osteoclast phenotype and increased bone resorption [105], Gal-1 and Gal-3 have emerged as major modulators of bone homeostasis. Indeed, Gal-3 showed the highest specificity for bone-related tissues among all the Galectin family members. Considered a marker of chondrogenic and osteogenic cell lineages, Gal-3 is expressed by chondrocytes, osteoclasts, and osteoblasts [106,107,108,109] and is able to interact with AGEs in osteoblast-like cells [110]. In addition, Gal-1 and Gal-3 are the main Gals expressed by bone marrow mesenchymal stem cells (BMSCs), able to differentiate in osteoblasts and regulate bone formation [111]. Worthy of note, Gal-1 and Gal-3 secretion from BMSCs seem to be involved in regulation of osteogenic differentiation and resolution of inflammation [112,113,114,115]. Compared to other Gals, both Gal-1 and Gal-3 can strictly modulate the T-cell immune response [116,117], whose dysregulation contributes to OP progression [118]. Moreover, Gal-1 and Gal-3 have also been reported to show the highest affinity for human factor VIII (FVIII) [119], closely related with mineralization and bone remodeling [120,121].

**Figure 4:**
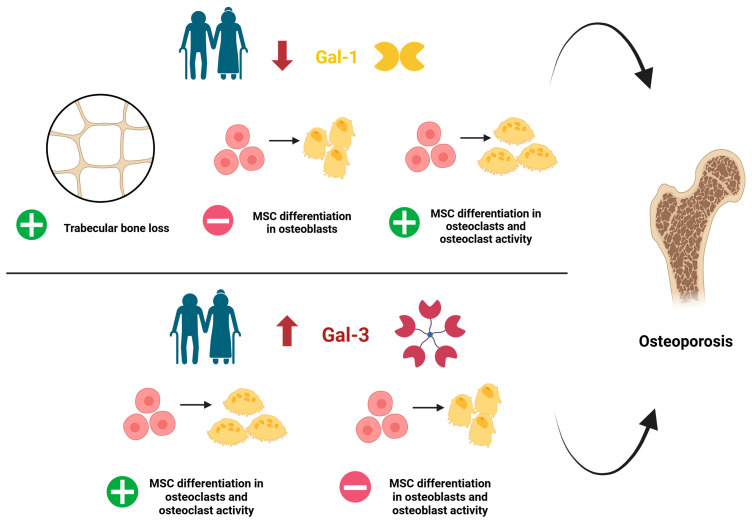
Dysregulation of Galectin 1 (Gal-1) and Galectin 3 (Gal-3) during ageing and main actions precipitating OP. Note that there is controversial evidence regarding the role of Gal-1 and Gal-3 in MSC osteoblast differentiation, as reported in Table 1. MSC: mesenchymal stem cells. +: increased; −: decreased. Created by Biorender.

To date, the evidence regarding the specific functions of Gal-1 and Gal-3 in bone homeostasis emerged from several preclinical settings is controversial. It is supported by limited clinical studies and necessitates further validation to elucidate the potential impact of Gal-1 and Gal-3 modulation in OP patients (Figure 4, Table 1).

A decline in Gal-1 serum levels in aged mice and humans was associated with age-related trabecular bone loss [122]. A study using Gal-1 knockout (KO) mice evidenced a decreased mineral density and alterations in trabecular microarchitecture in aged animals [112]. Moreover, BMSCs isolated from femur and tibia of Gal-1 KO mice exhibited a reduced differentiation into osteoblasts, partially restored by Gal-1 supplementation [112]. This latter evidence was lately confirmed by Takeuchi et al. (2024), who reported that both human and mouse BMSCs treated with Gal-1 showed a reduced osteoclast formation and bone resorption activity [123], suggesting a positive role for Gal-1 in regulating bone turnover. Furthermore, the equine BMSCs cultured in inflammatory conditions showed reduced Gal-1 levels: this could limit their intra-articular repair properties, due to a reduced differentiation into osteoblasts [114]. On the contrary, a negative role of Gal-1 in bone homeostasis has been suggested by an in vitro study reporting a decreased expression of osteocalcin [124], a key marker of osteoblast maturation [129], in BMSCs treated with Gal-1. This limited matrix mineralization.

Concerning Gal-3, the literature mainly describes a negative role in bone homeostasis (Table 1). Indeed, in response to high AGEs levels, Gal-3 levels increased in osteoblast-like cells [110] and inhibited human osteoblast differentiation by modulating the Notch signaling pathway [125]. Gal-3 also promoted osteoclast differentiation, thus influencing the extent of bone resorption [125]. In line with these data, Gal-3 inhibition in human fetal osteoblast cell line hFOB 1.19 promoted the proliferation and differentiation of osteoblasts, improving bone mineralization [126]. Similarly, Gal-3 KO mice exhibited increased osteoblastogenesis, resulting in preserved or increased bone mass [127]. Conversely, a positive role of Gal-3 as a novel regulator of osteoblast–osteoclast interaction has been recently proposed by Simon et al. (2017), who identified the secretion of Gal-3 by osteoblasts as a novel mechanism to control osteoclastogenesis and to maintain trabecular bone homeostasis [128].

### 3.2. Contribution of Gal-1 and Gal-3 to Secondary OP

Gal-1 and Gal-3 implication has been also underlined in several inflammaging-related pathologies contributing to OP, such as diabetes, obesity, and rheumatoid arthritis. In this regard, Gal-3 seems to induce inflammation and death of β-cells in pancreatic islets in patients with type 2 diabetes mellitus [130], with a specific role in the progression of prediabetes to diabetes stage [130] and diabetic nephropathy [131]. Conversely, Gal-1 is upregulated in the early stages of diabetic retinopathy (DR) and in its progression, while it is downregulated in the ocular microenvironment of non-retinopathic diabetic patients [79]. Furthermore, recent studies have highlighted the involvement of Gal-1 in liver and cardiac fibrosis induced by chronic diabetes [88,89]. Elevated Gal-1 and Gal-3 levels have been also reported in obese subjects [132]. Gals are also involved in arthritis, an inflammatory joint disease classified in rheumatoid arthritis (RA) and osteoarthritis (OA) [133,134,135]. Gal-1, often upregulated in RA, has been shown to positively correlate with markers of inflammation such as erythrocyte sedimentation rate and disease activity scores [136]. Its elevated levels may contribute to the regulation of immune responses in RA, potentially exerting anti-inflammatory effects in response to certain treatments [137].

Gal-3 levels are higher in RA than in OA, with Gal-3 substantially expressed and released from the inflamed synovial membrane in RA patients. These can trigger the release of pro-inflammatory cytokines and chemokines from rheumatoid fibrocyte-like synoviocytes [137,138,139]. Furthermore, Gal-3 plays a role in tissue damage through promoting the production of matrix metalloproteinases (MMPs), which contributes to joint degradation.

All over, these observations could lead to considering both Gal-1 and Gal-3 among the factors associated with the inflammaging alterations. Therefore, the ability to selectively modulate Gal-1 and Gal-3 may pave the way for a new therapeutic tool for bone health.

## 4. Perspective of Gal-1/Gal-3 Modulation in OP

To date, no specific Gal-1 and Gal-3 modulators have been tested in OP. Indeed, in OP animal models, the inhibition of Gal-1 and Gal-3 has been obtained by gene silencing [112,127,128]. However, several Gal inhibitors have been developed, such as tricyclic carbohydrate–benzene hybrids, natural carbohydrate-derived ligands, iron-containing glycomimetics based on lactose scaffolds, and thiodigalactosides (TDG) [140], or identified. Interestingly, these compounds are able to target several processes strictly related to inflammation and senescence. This suggests a potential benefit for their specific application in OP driven by inflammaging, in relation to the nonspecific use of bisphosphonates and denosumab in OP caused by post-menopausal syndrome, use of glucocorticoids, or secondary OP.

To this regard, Gal-3 expression seems to be reduced in preclinical models of atherosclerosis by melatonin treatment, leading to decreased inflammation and improved autophagy in macrophages [141]. In cardiac settings, Gal-3 inhibition has been obtained by the increase in the antioxidant protein peroxiredoxin-4 [142] and by the modified citrus pectin [143], with the consequent reduction in oxidative stress and inflammation. Of interest, the Gal-3 inhibitor G3P-01 (a pectin present in fruits and vegetables) has been orally administered for 4 months in aged men and women with elevated serum Gal-3 levels, as a nutritional dietary supplement aimed to favor a healthy aging process (NCT06398821). Therefore, from these preclinical and clinical studies, Gal-3 inhibition could emerge as a useful strategy aimed to specifically target several contributors to inflammaging-driven OP such as the low-grade inflammatory state, the oxidative stress, the ageing processes, and also the deleterious effects of reduced authophagy [144,145]. Indeed, an upregulated autophagic process favors the homeostasis of osteoblasts by promoting their survival (through reduced oxidative stress levels) and mineralization [146,147], overall improving trabecular bone mass and bone formation [148,149,150,151].

Regarding the other Gal-inhibitors under clinical investigation, the selective Gal-3 inhibitors GR-MD-02 (8 mg/kg) (NCT03809052, NCT04607655) or GB1211 (10 and 100 mg/kg) were administered orally, twice daily for 12 weeks, to patients with non-alcoholic steatohepatitis (NASH) (NCT02421094). A phase 2 clinical study is also ongoing in patients with idiopathic pulmonary fibrosis in order to evaluate the efficacy, pharmacokinetics, and pharmacodynamics of another Gal-3 inhibitor, GB0139, previously known as TD139, inhaled in the form of dry powder [152]. Worthy of note, a potential link between deteriorated bone health and fibrosis has been recently highlighted. Indeed, pulmonary fibrosis has been proposed as a risk factor for OP incidence in senile patients, showing a markedly reduced bone mineral density [153]. An attenuated bone architecture and a decreased bone density have been found also in aged patients affected by liver fibrosis [154,155]. Moreover, since fibrosis is a process strictly interrelated with senescence and inflammation [156,157,158], the results of the clinical studies assessing the efficacy and safety profile of Gal-3 inhibitors in fibrotic diseases could be useful also if translated to OP induced by inflammaging. Of interest, a specific anti-fibrotic effect has been shown also by the selective Gal-1 inhibitor OTX008 (or calixarene 0118) in human retinal pigment epithelium cells (ARPE-19) exposed to high glucose at different time-points, as a model of DR. The inhibition of Gal-1 by OTX008 preserved the integrity and functionality of retinal cells by reducing diabetes-induced fibrotic process [159]. A similar effect was obtained in an animal model of cardiac and liver fibrosis induced by chronic diabetes [88,89]. Although no clinical studies aimed to assess the anti-fibrotic effects of OTX008 has been conducted, a Phase I clinical study has been reported to test OTX008 subcutaneously administered (65 mg/day, daily, for 3 weeks) in patients with advanced solid tumors (NCT01724320). This starting for the preclinical evidence shows a reduction in tumor angiogenesis exerted by OTX008 through the inhibition of endothelial cell migration and the reduction in antitumor immune responses by the modulation of the tumor microenvironment [160,161]. Since elevated levels of both Gal-1 and Gal-3 are exhibited by patients with osteosarcoma [162,163,164,165], and long-term survivors of osteosarcoma show higher risks for OP prevalence and OF [166,167], a multifactorial approach aimed to target these Gals in osteoporotic patients with osteosarcoma, whose progression is favored by a state of chronic inflammation and aging/senescence-induced genes [168,169], could be hypothesized as an innovative therapeutic approach.

However, the clinical application of Gal-1 and Gal-3 inhibitors in inflammaging-related OP could be affected by some limitations, such as limited oral bioavailability, poor pharmacokinetic profiles, limited selectivity, challenges in assessing their affinity, and also in reaching specific target cells [140,170].

From the Gal-1 and Gal-3 activation point of view, their expressions have been increased only by specific gene promoters in cancer settings [171,172,173] and, to our knowledge, no specific activators have been tested either in OP or different diseases.

## 5. Conclusions

To date, there are contradictory preclinical data regarding the role of Gal-1 and Gal-3 in bone homeostasis, along with the limited availability of clinical studies and the absence of Gal specific modulator for bone tissue.

However, due to the several pieces of evidence highlighting the role of Gal-1 and Gal-3 in the modulation of inflammation, aging, and senescence, the need to approach Gal-1 and Gal-3 as novel targets of inflammaging-driven OP seems to be necessary. This could be achieved by improving the oral availability of the Gal-inhibitors already developed; by identifying the best candidate in terms of affinity, selectivity, and toxicity in OP preclinical settings; and by translating its potential application in OP patients by evaluating its pharmacokinetic profile, safety, and efficacy.

## Figures and Tables

**Table 1:** Preclinical and clinical studies on Gal-1 and Gal-3 in OP settings.

Reference	Study	Experimental Setting	Treatment	Main Results
Xu et al., 2021 [122]	In vivo Clinical	Aged male Balb/c and C57BL/6 mice Aged osteoporotic patients	/ /	Serum Gal-1 was reduced in aged mice and osteoporotic patients. Gal-1 decline was associated with trabecular bone mass loss in both preclinical and clinical settings
Chen et al., 2022 [112]	In vivo In vitro	Aged and young Gal-1 KO mice BMSCs isolated from femur and tibia of Gal-1 KO mice	/ Gal-1 0.5 μg/mL for 48 h	Deletion of Gal-1 in mice resulted in bone loss, due to a reduced ability of BMSCs to differentiate into osteoblasts. This was more evident in aged mice compared to young ones. In vitro, Gal-1 facilitated the differentiation of BMSCs into osteoblasts
Takeuchi et al., 2024 [123]	In vitro	Osteoclasts differentiated from Human PBMCs Osteoclasts differentiated from Raw 264.7	On both cell types: Recombinant Gal-1 protein, 10 µg/mL for 14 days	Recombinant Gal-1 inhibited osteoclast formation and bone resorption activity
Andersen et al., 2003 [124]	In vitro	Human BMSCs	Gal-1 recombinant (10–1000 ng/mL)	Gal-1 reduced Osteocalcin expression, suggesting a reduction in HBMSC differentiation in osteoblasts
Mercer et al., 2004 [110]	In vitro	MC3T3E1	100–200 microg/mL AGEs-BSA	Intracellular Gal-3 was increased by AGEs
Nakajima et al., 2016 [125]	In vitro	Raw 264.7 Human osteoclast precursors hFOB1.19	Cells exposed to full-length and cleaved Gal-3 secreted from breast and prostate cancer cells	Gal-3, through its interaction with the protein myosin-2A, promoted osteoclast differentiation. Furthermore, the cleaved Gal-3 influenced the extent of bone resorption
Nakajima et al., 2014 [126]	In vitro	hFOB1.19	Recombinant human Gal-3, 1.6 µM, every 3 to 4 days, for 3 weeks Lactose (75 mM), a sugar Gal-3 inhibitor	Gal-3 inhibited osteoblast differentiation through the Notch signaling pathway, and impaired bone formation by reducing the expression of genes implicated in osteoblastic differentiation, such as *RUNX2*, *SP7*, *ALPL*, *COL1A1*. Gal-3 inhibition promoted hFOB1.19 proliferation and differentiation
Maupin et al., 2018 [127]	In vivo	Gal-3 KO mice	/	Gal-3 KO mice exhibited preserved or enhanced bone mass, due to increased osteoblastogenesis.
Simon et al., 2017 [128]	In vivo In vitro	Gal-3 KO mice Osteoclasts and osteoblasts differentiated from Gal-3 KO BMSCs	/ /	Gal-3 KO mice exhibited elevated osteoclast numbers and a lowered trabecular bone volume. Gal-3 secreted by osteoblasts inhibited osteoclast formation

AGEs: Advanced Glycation End products; ALPL: Alkaline phosphatase; BMSCs: Bone Marrow Stromal Cells; BSA: Bovin Serum Albumin; COL1A1: Collagen type I alpha 1; Gal-1: Galectin 1; Gal-3: Galectin 3; hFOB1.19: human fetal osteoblastic cells; KO: knockout; MC3T3E1: mouse calvaria-derived osteoblasts; PBMCs: Peripheral Blood Mononuclear Cells; Raw 264.7: mouse osteoclast precursors; RUNX2: RUNX family transcription factor; SP7: SP7 transcription factor.

## Data Availability

Not applicable.

## Abbreviations

The following abbreviations are used in this manuscript:

AAS	anabolic agents
AD	Alzheimer’s disease
AGEs	advanced end products
ALPL	alkaline phosphatase
ARPE	human retinal pigment epithelium cells
BSA	bovine serum albumin
BMSCs	bone marrow mesenchymal stem cells
COL1A1	collagen type I alpha 1
COVID-19	Coronavirus disease 2019
DR	diabetic retinopathy
EAE	experimental autoimmune encephalomyelitis
FVIII	human factor VIII
Gal(s)	galectin(s)
Gal-(1-15)	galectin (1-15)
HRT	hormone Replacement Therapy
IGF	insulin-like growth factors
IL	interleukin
MMPs	metalloproteinases
MSCs	mesenchymal stem cells
OF	osteoporotic fractures
OP	osteoporosis
OA	osteoarthritis OA
OTX008	calixarene 0118
PTH	parathyroid hormone
RA	rheumatoid arthritis
RANKL	RANK ligand
ROS	reactive oxygen species
RUNX	RUNX family transcription factor
SERMS	selective estrogen receptor modulators
SP7	SP7 transcription factor
TDGs	thiodigalactosides
TGF-β	transforming growth factor-β
Th(1,2,17)	T helper (1,2,17)

## References

[1] Lane J.M., Russell L., Khan S.N. (2000). Osteoporosis. Clin. Orthop. Relat. Res..

[2] Rinonapoli G., Pace V., Ruggiero C., Ceccarini P., Bisaccia M., Meccariello L., Caraffa A. (2021). Obesity and Bone: A Complex Relationship. Int. J. Mol. Sci..

[3] Amarnath S.S., Kumar V., Das S.L. (2023). Classification of Osteoporosis. Indian J. Orthop..

[4] Srivastava M., Deal C. (2002). Osteoporosis in Elderly: Prevention and Treatment. Clin. Geriatr. Med..

[5] Freeman C., Tennyson J., Priscilla A.S. (2025). Genetic Variants of Vitamin D, Estrogen α, Parathyroid and Collagen Type I Alpha Receptor Gene and Its Influence on Circulating Serum Osteocalcin in Postmenopausal Osteoporosis: A Cohort Study. Nucleus.

[6] Mundy G.R., Shapiro J.L., Bandelin J.G., Canalis E.M., Raisz L.G. (1976). Direct Stimulation of Bone Resorption by Thyroid Hormones. J. Clin. Investig..

[7] Ala M., Jafari R.M., Dehpour A.R. (2020). Diabetes Mellitus and Osteoporosis Correlation: Challenges and Hopes. Curr. Diabetes Rev..

[8] Westhovens R., Dequeker J. (2000). Rheumatoid Arthritis and Osteoporosis. Z. Für Rheumatol..

[9] Lips P. (2007). Vitamin D Status and Nutrition in Europe and Asia. J. Steroid Biochem. Mol. Biol..

[10] Ohlhorst S.D., Russell R., Bier D., Klurfeld D.M., Li Z., Mein J.R., Milner J., Ross A.C., Stover P., Konopka E. (2013). Nutrition Research to Affect Food and a Healthy Lifespan. Adv. Nutr..

[11] Anderson J.J.B., Rondano P., Holmes A. (1996). Roles of Diet and Physical Activity in the Prevention of Osteoporosis. Scand. J. Rheumatol..

[12] Zavatta G., Clarke B.L. (2021). Glucocorticoid- and Transplantation-Induced Osteoporosis. Endocrinol. Metab. Clin. N. Am..

[13] Adami G., Fassio A., Gatti D., Viapiana O., Benini C., Danila M.I., Saag K.G., Rossini M. (2022). Osteoporosis in 10 Years Time: A Glimpse into the Future of Osteoporosis. Ther. Adv. Musculoskelet. Dis..

[14] Miller P.D. (2016). Management of Severe Osteoporosis. Expert Opin. Pharmacother..

[15] Odén A., McCloskey E.V., Johansson H., Kanis J.A. (2013). Assessing the Impact of Osteoporosis on the Burden of Hip Fractures. Calcif. Tissue Int..

[16] Verhaar H.J.J. (2008). Behandeling van Osteoporose Bij Ouderen: Wat Is de Evidence?. TGG.

[17] Adachi J.D., Ioannidis G., Berger C., Joseph L., Papaioannou A., Pickard L., Papadimitropoulos E.A., Hopman W., Poliquin S., Prior J.C. (2001). The Influence of Osteoporotic Fractures on Health-Related Quality of Life in Community-Dwelling Men and Women across Canada. Osteoporos. Int..

[18] Tarride J.-É., Adachi J.D., Brown J.P., Schemitsch E., Slatkovska L., Burke N. (2021). Incremental Costs of Fragility Fractures: A Population-Based Matched -Cohort Study from Ontario, Canada. Osteoporos. Int..

[19] Zeng Q., Li N., Wang Q., Feng J., Sun D., Zhang Q., Huang J., Wen Q., Hu R., Wang L. (2019). The Prevalence of Osteoporosis in China, a Nationwide, Multicenter DXA Survey. J. Bone Miner. Res..

[20] Jaleel A., Saag K.G., Danila M.I. (2018). Improving Drug Adherence in Osteoporosis: An Update on More Recent Studies. Ther. Adv. Musculoskelet. Dis..

[21] Sozen T., Ozisik L., Basaran N.C. (2017). An Overview and Management of Osteoporosis. Eur. J. Rheumatol..

[22] Kenkre J., Bassett J. (2018). The Bone Remodelling Cycle. Ann. Clin. Biochem..

[23] Seeman E., Delmas P.D. (2006). Bone Quality—The Material and Structural Basis of Bone Strength and Fragility. N. Engl. J. Med..

[24] Matsuoka K., Park K., Ito M., Ikeda K., Takeshita S. (2014). Osteoclast-Derived Complement Component 3a Stimulates Osteoblast Differentiation. J. Bone Miner. Res..

[25] Patel R.H., Lyles K.W., Duque G., Kiel D.P. (2009). Senile Osteoporosis as a Geriatric Syndrome. Osteoporosis in Older Persons.

[26] Huidrom S., Beg M.A., Masood T. (2021). Post-Menopausal Osteoporosis and Probiotics. Curr. Drug Targets.

[27] Curtis E., Litwic A., Cooper C., Dennison E. (2015). Determinants of Muscle and Bone Aging. J. Cell. Physiol..

[28] Föger-Samwald U., Kerschan-Schindl K., Butylina M., Pietschmann P. (2022). Age Related Osteoporosis: Targeting Cellular Senescence. Int. J. Mol. Sci..

[29] Bi J., Zhang C., Lu C., Mo C., Zeng J., Yao M., Jia B., Liu Z., Yuan P., Xu S. (2024). Age-Related Bone Diseases: Role of Inflammaging. J. Autoimmun..

[30] Lawrence M., Goyal A., Pathak S., Ganguly P. (2024). Cellular Senescence and Inflammaging in the Bone: Pathways, Genetics, Anti-Aging Strategies and Interventions. Int. J. Mol. Sci..

[31] Chandra A., Rajawat J. (2021). Skeletal Aging and Osteoporosis: Mechanisms and Therapeutics. Int. J. Mol. Sci..

[32] Jilka R.L., Takahashi K., Munshi M., Williams D.C., Roberson P.K., Manolagas S.C. (1998). Loss of Estrogen Upregulates Osteoblastogenesis in the Murine Bone Marrow. Evidence for Autonomy from Factors Released during Bone Resorption. J. Clin. Investig..

[33] Almeida M., Laurent M.R., Dubois V., Claessens F., O’Brien C.A., Bouillon R., Vanderschueren D., Manolagas S.C. (2017). Estrogens and Androgens in Skeletal Physiology and Pathophysiology. Physiol. Rev..

[34] Carter P., Schipani E. (2006). The Roles of Parathyroid Hormone and Calcitonin in Bone Remodeling: Prospects for Novel Therapeutics. EMIDDT.

[35] Abiri B., Vafa M., Guest P.C. (2020). Vitamin D and Muscle Sarcopenia in Aging. Clinical and Preclinical Models for Maximizing Healthspan.

[36] Giustina A., Bouillon R., Dawson-Hughes B., Ebeling P.R., Lazaretti-Castro M., Lips P., Marcocci C., Bilezikian J.P. (2022). Vitamin D in the Older Population: A Consensus Statement. Endocrine.

[37] Matikainen N., Pekkarinen T., Ryhänen E.M., Schalin-Jäntti C. (2021). Physiology of Calcium Homeostasis. Endocrinol. Metab. Clin. N. Am..

[38] Marzetti E., Leeuwenburgh C. (2006). Skeletal Muscle Apoptosis, Sarcopenia and Frailty at Old Age. Exp. Gerontol..

[39] Dao T., Green A.E., Kim Y.A., Bae S.-J., Ha K.-T., Gariani K., Lee M., Menzies K.J., Ryu D. (2020). Sarcopenia and Muscle Aging: A Brief Overview. Endocrinol. Metab..

[40] Sindel D. (2023). Osteoporosis: Spotlight on Current Approaches to Pharmacological Treatment. Turk. J. Phys. Med. Rehabil..

[41] Iolascon G., Moretti A., Toro G., Gimigliano F., Liguori S., Paoletta M. (2020). Pharmacological Therapy of Osteoporosis: What’s New?. CIA.

[42] Drake M.T., Clarke B.L., Khosla S. (2008). Bisphosphonates: Mechanism of Action and Role in Clinical Practice. Mayo Clin. Proc..

[43] Bilezikian J.P. (2009). Efficacy of Bisphosphonates in Reducing Fracture Risk in Postmenopausal Osteoporosis. Am. J. Med..

[44] Qayoom I., Raina D.B., Širka A., Tarasevičius Š., Tägil M., Kumar A., Lidgren L. (2018). Anabolic and Antiresorptive Actions of Locally Delivered Bisphosphonates for Bone Repair: A Review. Bone Jt. Res..

[45] Black D.M., Delmas P.D., Eastell R., Reid I.R., Boonen S., Cauley J.A., Cosman F., Lakatos P., Leung P.C., Man Z. (2007). Once-Yearly Zoledronic Acid for Treatment of Postmenopausal Osteoporosis. N. Engl. J. Med..

[46] Kennel K.A., Drake M.T. (2009). Adverse Effects of Bisphosphonates: Implications for Osteoporosis Management. Mayo Clin. Proc..

[47] Curry S.J., Krist A.H., Owens D.K., Barry M.J., Caughey A.B., Davidson K.W., Doubeni C.A., Epling J.W., Kemper A.R., US Preventive Services Task Force (2018). Screening for Osteoporosis to Prevent Fractures: US Preventive Services Task Force Recommendation Statement. JAMA.

[48] Miller P.D. (2011). The Kidney and Bisphosphonates. Bone.

[49] Toro G., Braile A., Liguori S., Moretti A., Landi G., Cecere A.B., Conza G., De Cicco A., Tarantino U., Iolascon G. (2023). The Role of the Fracture Liaison Service in the Prevention of Atypical Femoral Fractures. Ther. Adv. Musculoskelet..

[50] Toro G., Ojeda-Thies C., Calabrò G., Toro G., Moretti A., Guerra G.M.-D., Caba-Doussoux P., Iolascon G. (2016). Management of Atypical Femoral Fracture: A Scoping Review and Comprehensive Algorithm. BMC Musculoskelet. Disord..

[51] Sutton E.E., Riche D.M. (2012). Denosumab, a RANK Ligand Inhibitor, for Postmenopausal Women with Osteoporosis. Ann. Pharmacother..

[52] Kendler D.L., Cosman F., Stad R.K., Ferrari S. (2022). Denosumab in the Treatment of Osteoporosis: 10 Years Later: A Narrative Review. Adv. Ther..

[53] Ogasawara T., Yoshimine Y., Kiyoshima T., Kobayashi I., Matsuo K., Akamine A., Sakai H. (2004). In Situ Expression of RANKL, RANK, Osteoprotegerin and Cytokines in Osteoclasts of Rat Periodontal Tissue. J. Periodontal Res..

[54] Reid I.R., Billington E.O. (2022). Drug Therapy for Osteoporosis in Older Adults. Lancet.

[55] Inderjeeth C.A., Inderjeeth D.C. (2024). The Use of Anabolic Agents in the Treatment of Osteoporosis: A Clinical Update. Curr. Opin. Endocrinol. Diabetes Obes..

[56] Sølling A.S.K., Harsløf T., Langdahl B. (2018). The Clinical Potential of Romosozumab for the Prevention of Fractures in Postmenopausal Women with Osteoporosis. Ther. Adv. Musculoskelet..

[57] Cosman F., Crittenden D.B., Adachi J.D., Binkley N., Czerwinski E., Ferrari S., Hofbauer L.C., Lau E., Lewiecki E.M., Miyauchi A. (2016). Romosozumab Treatment in Postmenopausal Women with Osteoporosis. N. Engl. J. Med..

[58] Saag K.G., Petersen J., Brandi M.L., Karaplis A.C., Lorentzon M., Thomas T., Maddox J., Fan M., Meisner P.D., Grauer A. (2017). Romosozumab or Alendronate for Fracture Prevention in Women with Osteoporosis. N. Engl. J. Med..

[59] Langdahl B.L., Libanati C., Crittenden D.B., Bolognese M.A., Brown J.P., Daizadeh N.S., Dokoupilova E., Engelke K., Finkelstein J.S., Genant H.K. (2017). Romosozumab (Sclerostin Monoclonal Antibody) versus Teriparatide in Postmenopausal Women with Osteoporosis Transitioning from Oral Bisphosphonate Therapy: A Randomised, Open-Label, Phase 3 Trial. Lancet.

[60] Liu Y., Cao Y., Zhang S., Zhang W., Zhang B., Tang Q., Li Z., Wu J. (2018). Romosozumab Treatment in Postmenopausal Women with Osteoporosis: A Meta-Analysis of Randomized Controlled Trials. Climacteric.

[61] Lewiecki E.M., Blicharski T., Goemaere S., Lippuner K., Meisner P.D., Miller P.D., Miyauchi A., Maddox J., Chen L., Horlait S. (2018). A Phase III Randomized Placebo-Controlled Trial to Evaluate Efficacy and Safety of Romosozumab in Men with Osteoporosis. J. Clin. Endocrinol. Metab..

[62] Bhatnagar A., Kekatpure A.L. (2022). Postmenopausal Osteoporosis: A Literature Review. Cureus.

[63] Patel H.K., Bihani T. (2018). Selective Estrogen Receptor Modulators (SERMs) and Selective Estrogen Receptor Degraders (SERDs) in Cancer Treatment. Pharmacol. Ther..

[64] Santoro N., Epperson C.N., Mathews S.B. (2015). Menopausal Symptoms and Their Management. Endocrinol. Metab. Clin. N. Am..

[65] Moshi M.R., Nicolopoulos K., Stringer D., Ma N., Jenal M., Vreugdenburg T. (2023). The Clinical Effectiveness of Denosumab (Prolia^®^) for the Treatment of Osteoporosis in Postmenopausal Women, Compared to Bisphosphonates, Selective Estrogen Receptor Modulators (SERM), and Placebo: A Systematic Review and Network Meta-Analysis. Calcif. Tissue Int..

[66] Francis R.M., Peacock M., Taylor G.A., Storer J.H., Nordin B.E.C. (1984). Calcium Malabsorption in Elderly Women with Vertebral Fractures: Evidence for Resistance to the Action of Vitamin D Metabolites on the Bowel. Clin. Sci..

[67] Muñoz M., Robinson K., Shibli-Rahhal A. (2020). Bone Health and Osteoporosis Prevention and Treatment. Clin. Obstet. Gynecol..

[68] Bertoldo F., Cianferotti L., Di Monaco M., Falchetti A., Fassio A., Gatti D., Gennari L., Giannini S., Girasole G., Gonnelli S. (2022). Definition, Assessment, and Management of Vitamin D Inadequacy: Suggestions, Recommendations, and Warnings from the Italian Society for Osteoporosis, Mineral Metabolism and Bone Diseases (SIOMMMS). Nutrients.

[69] Mehta N., Malootian A., Gilligan J. (2003). Calcitonin for Osteoporosis and Bone Pain. Curr. Pharm. Des..

[70] Pino A.M., Rosen C.J., Rodríguez J.P. (2012). In Osteoporosis, Differentiation of Mesenchymal Stem Cells (MSCs) Improves Bone Marrow Adipogenesis. Biol. Res..

[71] Indran I.R., Liang R.L.Z., Min T.E., Yong E.-L. (2016). Preclinical Studies and Clinical Evaluation of Compounds from the Genus Epimedium for Osteoporosis and Bone Health. Pharmacol. Ther..

[72] Meng F.-H., Li Y.-B., Xiong Z.-L., Jiang Z.-M., Li F.-M. (2005). Osteoblastic Proliferative Activity of Epimedium Brevicornum Maxim. Phytomedicine.

[73] Streicher C., Heyny A., Andrukhova O., Haigl B., Slavic S., Schüler C., Kollmann K., Kantner I., Sexl V., Kleiter M. (2017). Estrogen Regulates Bone Turnover by Targeting RANKL Expression in Bone Lining Cells. Sci. Rep..

[74] Siu W., Wong H., Lau C., Shum W., Wong C., Gao S., Fung K., Lau C.B., Hung L., Ko C. (2013). The Effects of an Antiosteoporosis Herbal Formula Containing Epimedii Herba, Ligustri Lucidi Fructus and Psoraleae Fructus on Density and Structure of Rat Long Bones Under Tail-Suspension, and Its Mechanisms of Action. Phytother. Res..

[75] Si Y., Li Y., Gu K., Yin H., Ma Y. (2024). Icariin Ameliorates Osteoporosis in Ovariectomized Rats by Targeting Cullin 3/Nrf2/OH Pathway for Osteoclast Inhibition. Biomed. Pharmacother..

[76] Liu R., Kang X., Xu L., Nian H., Yang X., Shi H., Wang X. (2015). Effect of the Combined Extracts of Herba Epimedii and Fructus Ligustri Lucidi on Sex Hormone Functional Levels in Osteoporosis Rats. Evid.-Based Complement. Altern. Med..

[77] Bai L., Liu Y., Zhang X., Chen P., Hang R., Xiao Y., Wang J., Liu C. (2023). Osteoporosis Remission via an Anti-Inflammaging Effect by Icariin Activated Autophagy. Biomaterials.

[78] Liu F.-T., Stowell S.R. (2023). The Role of Galectins in Immunity and Infection. Nat. Rev. Immunol..

[79] Hermenean A., Oatis D., Herman H., Ciceu A., D’Amico G., Trotta M.C. (2022). Galectin 1—A Key Player between Tissue Repair and Fibrosis. Int. J. Mol. Sci..

[80] Dings R., Miller M., Griffin R., Mayo K. (2018). Galectins as Molecular Targets for Therapeutic Intervention. Int. J. Mol. Sci..

[81] Cooper D., Norling L.V., Perretti M. (2008). Novel Insights into the Inhibitory Effects of Galectin-1 on Neutrophil Recruitment under Flow. J. Leukoc. Biol..

[82] Hsu D.K., Yang R.-Y., Pan Z., Yu L., Salomon D.R., Fung-Leung W.-P., Liu F.-T. (2000). Targeted Disruption of the Galectin-3 Gene Results in Attenuated Peritoneal Inflammatory Responses. Am. J. Pathol..

[83] Colnot C., Ripoche M.A., Milon G., Montagutelli X., Crocker P.R., Poirier F. (1998). Maintenance of Granulocyte Numbers during Acute Peritonitis Is Defective in Galectin-3-null Mutant Mice. Immunology.

[84] Offner H., Celnik B., Bringman T.S., Casentini-Borocz D., Nedwin G.E., Vandenbark A.A. (1990). Recombinant Human β-Galactoside Binding Lectin Suppresses Clinical and Histological Signs of Experimental Autoimmune Encephalomyelitis. J. Neuroimmunol..

[85] Santucci L., Fiorucci S., Rubinstein N., Mencarelli A., Palazzetti B., Federici B., Rabinovich G.A., Morelli A. (2003). Galectin-1 Suppresses Experimental Colitis in Mice. Gastroenterology.

[86] Baum L.G., Blackall D.P., Arias-Magallano S., Nanigian D., Uh S.Y., Browne J.M., Hoffmann D., Emmanouilides C.E., Territo M.C., Baldwin G.C. (2003). Amelioration of Graft versus Host Disease by Galectin-1. Clin. Immunol..

[87] Toscano M.A., Commodaro A.G., Ilarregui J.M., Bianco G.A., Liberman A., Serra H.M., Hirabayashi J., Rizzo L.V., Rabinovich G.A. (2006). Galectin-1 Suppresses Autoimmune Retinal Disease by Promoting Concomitant Th2- and T Regulatory-Mediated Anti-Inflammatory Responses. J. Immunol..

[88] Trotta M.C., Herman H., Ciceu A., Mladin B., Rosu M., Lepre C.C., Russo M., Bácskay I., Fenyvesi F., Marfella R. (2023). Chrysin-Based Supramolecular Cyclodextrin-Calixarene Drug Delivery System: A Novel Approach for Attenuating Cardiac Fibrosis in Chronic Diabetes. Front. Pharmacol..

[89] Balta C., Herman H., Ciceu A., Lepre C.C., Mladin B., Rosu M., Oatis D., Russo M., Peteu V.E., Gherghiceanu M. (2024). Chrysin-Loaded Calixarene-Cyclodextrin Ternary Drug Delivery System Inhibits TGF-β and Galectin-1 Mediated Pathways in Diabetic Liver Fibrosis. Biochem. Pharmacol..

[90] Loser K., Sturm A., Voskort M., Kupas V., Balkow S., Auriemma M., Sternemann C., Dignass A.U., Luger T.A., Beissert S. (2009). Galectin-2 Suppresses Contact Allergy by Inducing Apoptosis in Activated CD8+ T Cells. J. Immunol..

[91] Zuberi R.I., Hsu D.K., Kalayci O., Chen H.-Y., Sheldon H.K., Yu L., Apgar J.R., Kawakami T., Lilly C.M., Liu F.-T. (2004). Critical Role for Galectin-3 in Airway Inflammation and Bronchial Hyperresponsiveness in a Murine Model of Asthma. Am. J. Pathol..

[92] Saegusa J., Hsu D.K., Chen H.-Y., Yu L., Fermin A., Fung M.A., Liu F.-T. (2009). Galectin-3 Is Critical for the Development of the Allergic Inflammatory Response in a Mouse Model of Atopic Dermatitis. Am. J. Pathol..

[93] Jiang H.-R., Al Rasebi Z., Mensah-Brown E., Shahin A., Xu D., Goodyear C.S., Fukada S.Y., Liu F.-T., Liew F.Y., Lukic M.L. (2009). Galectin-3 Deficiency Reduces the Severity of Experimental Autoimmune Encephalomyelitis. J. Immunol..

[94] Oatis D., Balta C., Herman H., Ciceu A., Simon-Repolski E., Mihu A.G., Lepre C.C., Russo M., Trotta M.C., D’Amico G. (2025). The Interplay between Lung Galectins and Pro-Fibrotic Markers in Post-COVID-19 Fibrogenesis: A Pilot Study. Life Sci..

[95] Hokama A., Mizoguchi E., Sugimoto K., Shimomura Y., Tanaka Y., Yoshida M., Rietdijk S.T., De Jong Y.P., Snapper S.B., Terhorst C. (2004). Induced Reactivity of Intestinal CD4+ T Cells with an Epithelial Cell Lectin, Galectin-4, Contributes to Exacerbation of Intestinal Inflammation. Immunity.

[96] Zhu C., Anderson A.C., Schubart A., Xiong H., Imitola J., Khoury S.J., Zheng X.X., Strom T.B., Kuchroo V.K. (2005). The Tim-3 Ligand Galectin-9 Negatively Regulates T Helper Type 1 Immunity. Nat. Immunol..

[97] Pricci F., Leto G., Amadio L., Iacobini C., Romeo G., Cordone S., Gradini R., Barsotti P., Liu F.-T., Di Mario U. (2000). Role of Galectin-3 as a Receptor for Advanced Glycosylation End Products. Kidney Int..

[98] Ji X., Jiang Z., Qiu Y., Yu J., Zhang Y., Wang J., Ye B., Huang Y., Gu W., Huang Y. (2023). High Blood Galectin-3 Level Associated with Risk of Frailty in Aging. Front. Endocrinol..

[99] Sanchis-Gomar F., Santos-Lozano A., Pareja-Galeano H., Garatachea N., Alis R., Fiuza-Luces C., Morán M., Emanuele E., Lucia A. (2016). Galectin-3, Osteopontin and Successful Aging. Clin. Chem. Lab. Med. (CCLM).

[100] Xue S., Lozinski B.M., Ghorbani S., Ta K., D’Mello C., Yong V.W., Dong Y. (2023). Elevated Galectin-3 Is Associated with Aging, Multiple Sclerosis, and Oxidized Phosphatidylcholine-Induced Neurodegeneration. J. Neurosci..

[101] Estevez F., Florencia S., Betazza C. (2022). Genetic Deletion of Galectin-3 Exacerbates Age-Related Myocardial Hypertrophy and Fibrosis in Mice. Cell. Physiol. Biochem..

[102] Mir M.Y., Legradi A. (2025). Sweet Aging: Glycocalyx and Galectins in CNS Aging and Neurodegenerative Disorders. Int. J. Mol. Sci..

[103] Kiss T., Mir Y., Stefancsik G., Ganbat G., Askarova A., Monostori E., Dulka K., Szebeni G.J., Nyúl-Tóth Á., Csiszár A. (2023). Galectin-1 as a Marker for Microglia Activation in the Aging Brain. Brain Res..

[104] Zhang G., Peng Q., Guo X., Pan L., Xiong M., Zhang X., Dai L., Zhang Z., Xiao T., He J. (2025). Microglia-derived Galectin-9 Drives Amyloid-β Pathology in Alzheimer’s Disease. Aging Cell.

[105] Roy M., Nguimbus L.M., Badiane P.Y., Goguen-Couture V., Degrandmaison J., Parent J.-L., Brunet M.A., Roux S. (2024). Galectin-8 Modulates Human Osteoclast Activity Partly through Isoform-Specific Interactions. Life Sci. Alliance.

[106] Iacobini C., Fantauzzi C.B., Pugliese G., Menini S. (2017). Role of Galectin-3 in Bone Cell Differentiation, Bone Pathophysiology and Vascular Osteogenesis. Int. J. Mol. Sci..

[107] Zhang Y., Li Z., Chen X. (2024). The role of galectin-3 in bone homeostasis: A review. Int. J. Biol. Macromol..

[108] Clevers H., Nusse R. (2012). Wnt/β-catenin signaling and disease. Cell.

[109] Duan P., Bonewald L.F. (2016). The role of the wnt/β-catenin signaling pathway in formation and maintenance of bone and teeth. Int. J. Biochem. Cell Biol..

[110] Mercer N., Ahmed H., McCarthy A.D., Etcheverry S.B., Vasta G.R., Cortizo A.M. (2004). AGE-R3/Galectin-3 Expression in Osteoblast-like Cells: Regulation by AGEs. Mol. Cell. Biochem..

[111] Gao Q., Wang L., Wang S., Huang B., Jing Y., Su J. (2022). Bone Marrow Mesenchymal Stromal Cells: Identification, Classification, and Differentiation. Front. Cell Dev. Biol..

[112] Chen Y., Liu Y., Zhang Y., Yu J., Tang L. (2022). Galectin-1 deletion in mice causes bone loss via impaired osteogenic differentiation potential of BMSCs. FASEB J..

[113] Ge X., Shi K., Hou J., Fu Y., Xiao H., Chi F., Xu J., Cai F., Bai C. (2021). Galectin-1 secreted by bone marrow-derived mesenchymal stem cells mediates anti-inflammatory responses in acute airway disease. Exp. Cell Res..

[114] Reesink H.L., Sutton R.M., Shurer C.R., Peterson R.P., Tan J.S., Su J., Paszek M.J., Nixon A.J. (2017). Galectin-1 and Galectin-3 Expression in Equine Mesenchymal Stromal Cells (MSCs), Synovial Fibroblasts and Chondrocytes, and the Effect of Inflammation on MSC Motility. Stem Cell Res. Ther..

[115] Xu L., Qian Z., Wang S., Wang R., Pu X., Yang B., Zhou Q., Du C., Chen Q., Feng Z. (2022). Galectin-3 Enhances Osteogenic Differentiation of Precursor Cells From Patients With Diffuse Idiopathic Skeletal Hyperostosis via Wnt/β-Catenin Signaling. J. Bone Min. Res..

[116] Deák M., Hornung Á., Novák J., Demydenko D., Szabó E., Czibula Á., Fajka-Boja R., Kriston-Pál É., Monostori É., Kovács L. (2015). Novel role for galectin-1 in T-cells under physiological and pathological conditions. Immunobiology.

[117] Gilson R.C., Gunasinghe S.D., Johannes L., Gaus K. (2019). Galectin-3 modulation of T-cell activation: Mechanisms of membrane remodelling. Prog. Lipid Res..

[118] Zhang W., Dang K., Huai Y., Qian A. (2020). Osteoimmunology: The Regulatory Roles of T Lymphocytes in Osteoporosis. Front. Endocrinol..

[119] O’Sullivan J.M., Jenkins P.V., Rawley O., Gegenbauer K., Chion A., Lavin M., Byrne B., O’Kennedy R., Preston R.J., Brophy T.M. (2016). Galectin-1 and Galectin-3 Constitute Novel-Binding Partners for Factor VIII. Arterioscler. Thromb. Vasc. Biol..

[120] Battafarano G., Lancellotti S., Sacco M., Rossi M., Terreri S., Di Gregorio J., Di Giuseppe L., D’Agostini M., Porzio O., Di Gennaro L. (2024). Effects of coagulation factors on bone cells and consequences of their absence in haemophilia a patients. Sci. Rep..

[121] Cadé M., Muñoz-Garcia J., Babuty A., Fouassier M., Heymann M.F., Monahan P.E., Heymann D. (2022). FVIII at the crossroad of coagulation, bone and immune biology: Emerging evidence of biological activities beyond hemostasis. Drug Discov. Today.

[122] Xu W., Ni C., Wang Y., Zheng G., Zhang J., Xu Y. (2021). Age-Related Trabecular Bone Loss Is Associated with a Decline in Serum Galectin-1 Level. BMC Musculoskelet. Disord..

[123] Takeuchi T., Oyama M., Tamura M., Arata Y., Hatanaka T. (2024). Reduced Form of Galectin-1 Suppresses Osteoclastic Differentiation of Human Peripheral Blood Mononuclear Cells and Murine RAW264 Cells In Vitro. Biomolecules.

[124] Andersen H., Jensen O.N., Moiseeva E.P., Eriksen E.F. (2003). A Proteome Study of Secreted Prostatic Factors Affecting Osteoblastic Activity: Galectin-1 Is Involved in Differentiation of Human Bone Marrow Stromal Cells. J. Bone Miner. Res..

[125] Nakajima K., Kho D.H., Yanagawa T., Harazono Y., Hogan V., Chen W., Ali-Fehmi R., Mehra R., Raz A. (2016). Galectin-3 Cleavage Alters Bone Remodeling: Different Outcomes in Breast and Prostate Cancer Skeletal Metastasis. Cancer Res..

[126] Nakajima K., Kho D.H., Yanagawa T., Harazono Y., Gao X., Hogan V., Raz A. (2014). Galectin-3 Inhibits Osteoblast Differentiation through Notch Signaling. Neoplasia.

[127] Maupin K.A., Weaver K., Bergsma A., Christie C., Zhong Z.A., Yang T., Williams B.O. (2018). Enhanced cortical bone expansion in Lgals3-deficient mice during aging. Bone Res..

[128] Simon D., Derer A., Andes F.T., Lezuo P., Bozec A., Schett G., Herrmann M., Harre U. (2017). Galectin-3 as a Novel Regulator of Osteoblast-Osteoclast Interaction and Bone Homeostasis. Bone.

[129] Nakamura A., Dohi Y., Akahane M., Ohgushi H., Nakajima H., Funaoka H., Takakura Y. (2009). Osteocalcin Secretion as an Early Marker of In Vitro Osteogenic Differentiation of Rat Mesenchymal Stem Cells. Tissue Eng. Part C Methods.

[130] Li Y., Li T., Zhou Z., Xiao Y. (2022). Emerging Roles of Galectin-3 in Diabetes and Diabetes Complications: A Snapshot. Rev. Endocr. Metab. Disord..

[131] Guo Y., Li L., Hu S. (2023). Circulating Galectin-3 Levels and Diabetic Nephropathy: A Systematic Review and Meta-Analysis. BMC Nephrol..

[132] Aksit M.Z., Demet Arslan F., Karakoyun I., Aydin C., Turgut E., Parildar H., Gokbalci U., Isbilen Basok B., Duman C., Emiroglu M. (2022). Galectin-3 Levels and Inflammatory Response in Patients Undergoing Bariatric Surgery. Cytokine.

[133] Osório J. (2016). Galectin-1 Damages Cartilage via Inflammation. Nat. Rev. Rheumatol..

[134] Triguero-Martínez A., Roy-Vallejo E., Tomero E.G., Montes N., Fuente H.D.L., Ortiz A.M., Castañeda S., Lamana A., González-Álvaro I. (2022). Galectin-1: A Potential Biomarker Differentiating between Early Rheumatoid Arthritis and Spondyloarthritis. J. Clin. Med..

[135] Mohammed A., Alshamarri T., Adeyeye T., Lazariu V., McNutt L.-A., Carpenter D.O. (2020). A Comparison of Risk Factors for Osteo- and Rheumatoid Arthritis Using NHANES Data. Prev. Med. Rep..

[136] Matsumoto H., Fujita Y., Asano T., Matsuoka N., Temmoku J., Sato S., Yashiro–Furuya M., Yokose K., Yoshida S., Suzuki E. (2021). Association between Inflammatory Cytokines and Immune–Checkpoint Molecule in Rheumatoid Arthritis. PLoS ONE.

[137] Mendez-Huergo S.P., Hockl P.F., Stupirski J.C., Maller S.M., Morosi L.G., Pinto N.A., Berón A.M., Musuruana J.L., Nasswetter G.G., Cavallasca J.A. (2019). Clinical Relevance of Galectin-1 and Galectin-3 in Rheumatoid Arthritis Patients: Differential Regulation and Correlation With Disease Activity. Front. Immunol..

[138] Toscano M.A., Martínez Allo V.C., Cutine A.M., Rabinovich G.A., Mariño K.V. (2018). Untangling Galectin-Driven Regulatory Circuits in Autoimmune Inflammation. Trends Mol. Med..

[139] Cedeno-Laurent F., Dimitroff C.J. (2012). Galectin-1 Research in T Cell Immunity: Past, Present and Future. Clin. Immunol..

[140] Laderach D.J., Compagno D. (2023). Inhibition of Galectins in Cancer: Biological Challenges for Their Clinical Application. Front. Immunol..

[141] Wang Z., Gao Z., Zheng Y., Kou J., Song D., Yu X., Dong B., Chen T., Yang Y., Gao X. (2023). Melatonin Inhibits Atherosclerosis Progression via Galectin-3 Downregulation to Enhance Autophagy and Inhibit Inflammation. J. Pineal Res..

[142] Ibarrola J., Arrieta V., Sádaba R., Martinez-Martinez E., Garcia-Peña A., Alvarez V., Fernández-Celis A., Gainza A., Santamaría E., Fernández-Irigoyen J. (2018). Galectin-3 down-Regulates Antioxidant Peroxiredoxin-4 in Human Cardiac Fibroblasts: A New Pathway to Induce Cardiac Damage. Clin. Sci..

[143] Mahmoud M.M., Hassan M.M., Elsayed H.E.-S., Fares A.E., Saber M.M., Rashed L.A., Abdelwahed O.M. (2025). Protective Effect of Galectin-3 Inhibitor against Cardiac Remodelling in an Isoprenaline-Induced Myocardial Infarction in Type 2 Diabetes. Arch. Physiol. Biochem..

[144] Livshits G., Kalinkovich A. (2022). Targeting Chronic Inflammation as a Potential Adjuvant Therapy for Osteoporosis. Life Sci..

[145] Zhang C., Li H., Li J., Hu J., Yang K., Tao L. (2023). Oxidative Stress: A Common Pathological State in a High-Risk Population for Osteoporosis. Biomed. Pharmacother..

[146] Yin X., Zhou C., Li J., Liu R., Shi B., Yuan Q., Zou S. (2019). Autophagy in Bone Homeostasis and the Onset of Osteoporosis. Bone Res..

[147] Nollet M., Santucci-Darmanin S., Breuil V., Al-Sahlanee R., Cros C., Topi M., Momier D., Samson M., Pagnotta S., Cailleteau L. (2014). Autophagy in Osteoblasts Is Involved in Mineralization and Bone Homeostasis. Autophagy.

[148] DeSelm C.J., Miller B.C., Zou W., Beatty W.L., van Meel E., Takahata Y., Klumperman J., Tooze S.A., Teitelbaum S.L., Virgin H.W. (2011). Autophagy Proteins Regulate the Secretory Component of Osteoclastic Bone Resorption. Dev. Cell.

[149] Liu F., Fang F., Yuan H., Yang D., Chen Y., Williams L., Goldstein S.A., Krebsbach P.H., Guan J.-L. (2013). Suppression of Autophagy by FIP200 Deletion Leads to Osteopenia in Mice through the Inhibition of Osteoblast Terminal Differentiation. J. Bone Miner. Res..

[150] Li H., Li D., Ma Z., Qian Z., Kang X., Jin X., Li F., Wang X., Chen Q., Sun H. (2018). Defective Autophagy in Osteoblasts Induces Endoplasmic Reticulum Stress and Causes Remarkable Bone Loss. Autophagy.

[151] Piemontese M., Onal M., Xiong J., Han L., Thostenson J.D., Almeida M., O’Brien C.A. (2016). Low Bone Mass and Changes in the Osteocyte Network in Mice Lacking Autophagy in the Osteoblast Lineage. Sci. Rep..

[152] Oatis D., Simon-Repolski E., Balta C., Mihu A., Pieretti G., Alfano R., Peluso L., Trotta M.C., D’Amico M., Hermenean A. (2022). Cellular and Molecular Mechanism of Pulmonary Fibrosis Post-COVID-19: Focus on Galectin-1, -3, -8, -9. Int. J. Mol. Sci..

[153] Xie Z., He Y., Sun Y., Lin Z., Yang M., Liu Q., Liu S. (2016). Association between Pulmonary Fibrosis and Osteoporosis in the Elderly People: A Case–Control Study. Medicine.

[154] Pan B., Zhao Y., Chen C., Cai J., Li K., Wang Y., Liu J. (2024). The Relationship between Advanced Liver Fibrosis and Osteoporosis in Type 2 Diabetes Patients with MAFLD. Endocrine.

[155] Soltani A., Aghakhani A., Dehghanbanadaki H., Majidi Z., Rezaei-Tavirani M., Shafiee G., Ostovar A., Mir Moeini S.A., Bandarian F., Larijani B. (2025). Association between Liver Fibrosis and Osteoporosis in Adults Aged 50 and Older: Insights from the Bushehr Elderly Health Program. J. Diabetes Metab. Disord..

[156] Venosa A. (2020). Senescence in Pulmonary Fibrosis: Between Aging and Exposure. Front. Med..

[157] Godoy M.C.X., Monteiro G.A., Moraes B.H., Macedo J.A., Gonçalves G.M.S., Gambero A. (2024). Addition of Polyphenols to Drugs: The Potential of Controlling “Inflammaging” and Fibrosis in Human Senescent Lung Fibroblasts In Vitro. Int. J. Mol. Sci..

[158] Torres-Machorro A.L., García-Vicente Á., Espina-Ordoñez M., Luis-García E., Negreros M., Herrera I., Becerril C., Toscano F., Cisneros J., Maldonado M. (2025). Update of Aging Hallmarks in Idiopathic Pulmonary Fibrosis. Cells.

[159] Trotta M.C., Petrillo F., Gesualdo C., Rossi S., Corte A.D., Váradi J., Fenyvesi F., D’Amico M., Hermenean A. (2022). Effects of the Calix [4]Arene Derivative Compound OTX008 on High Glucose-Stimulated ARPE-19 Cells: Focus on Galectin-1/TGF-β/EMT Pathway. Molecules.

[160] Liu F.-T., Rabinovich G.A. (2005). Galectins as Modulators of Tumour Progression. Nat. Rev. Cancer.

[161] Astorgues-Xerri L., Riveiro M.E., Tijeras-Raballand A., Serova M., Rabinovich G.A., Bieche I., Vidaud M., de Gramont A., Martinet M., Cvitkovic E. (2014). OTX008, a Selective Small-Molecule Inhibitor of Galectin-1, Downregulates Cancer Cell Proliferation, Invasion and Tumour Angiogenesis. Eur. J. Cancer.

[162] Gomez-Brouchet A., Mourcin F., Gourraud P.-A., Bouvier C., De Pinieux G., Le Guelec S., Brousset P., Delisle M.-B., Schiff C. (2010). Galectin-1 Is a Powerful Marker to Distinguish Chondroblastic Osteosarcoma and Conventional Chondrosarcoma. Hum. Pathol..

[163] Park G.B., Kim D.-J., Kim Y.-S., Lee H.-K., Kim C.W., Hur D.Y. (2015). Silencing of Galectin-3 Represses Osteosarcoma Cell Migration and Invasion through Inhibition of FAK/Src/Lyn Activation and β-Catenin Expression and Increases Susceptibility to Chemotherapeutic Agents. Int. J. Oncol..

[164] Zhou X., Jing J., Peng J., Mao W., Zheng Y., Wang D., Wang X., Liu Z., Zhang X. (2014). Expression and Clinical Significance of Galectin-3 in Osteosarcoma. Gene.

[165] Kaur P., Kotru S., Singh S., Munshi A. (2022). miRNA Signatures in Diabetic Retinopathy and Nephropathy: Delineating Underlying Mechanisms. J. Physiol. Biochem..

[166] Lim J.S., Kim D.H., Lee J.A., Kim D.H., Cho J., Cho W.H., Lee S.-Y., Jeon D.-G. (2013). Young Age at Diagnosis, Male Sex, and Decreased Lean Mass Are Risk Factors of Osteoporosis in Long-Term Survivors of Osteosarcoma. J. Pediatr. Hematol./Oncol..

[167] Bellini G., Pinto D.D., Tortora C., Manzo I., Punzo F., Casale F., Rossi F. (2017). The Role of Mifamurtide in Chemotherapy-Induced Osteoporosis of Children with Osteosarcoma. Curr. Cancer Drug Targets.

[168] Mori T., Sato Y., Miyamoto K., Kobayashi T., Shimizu T., Kanagawa H., Katsuyama E., Fujie A., Hao W., Tando T. (2014). TNFα promotes osteosarcoma progression by maintaining tumor cells in an undifferentiated state. Oncogene.

[169] Lv Y., Wu L., Jian H., Zhang C., Lou Y., Kang Y., Hou M., Li Z., Li X., Sun B. (2022). Identification and characterization of aging/senescence-induced genes in osteosarcoma and predicting clinical prognosis. Front. Immunol..

[170] Zetterberg F.R., MacKinnon A., Brimert T., Gravelle L., Johnsson R.E., Kahl-Knutson B., Leffler H., Nilsson U.J., Pedersen A., Peterson K. (2022). Discovery and Optimization of the First Highly Effective and Orally Available Galectin-3 Inhibitors for Treatment of Fibrotic Disease. J. Med. Chem..

[171] Park K.-S., Kim J.-S. (2006). Engineering of GAL1 Promoter-Driven Expression System with Artificial Transcription Factors. Biochem. Biophys. Res. Commun..

[172] Deng J., Wu Y., Zheng Z., Chen N., Luo X., Tang H., Keasling J.D. (2021). A Synthetic Promoter System for Well-Controlled Protein Expression with Different Carbon Sources in Saccharomyces Cerevisiae. Microb. Cell Fact..

[173] Ahmed H., Cappello F., Rodolico V., Vasta G.R. (2009). Evidence of Heavy Methylation in the Galectin 3 Promoter in Early Stages of Prostate Adenocarcinoma: Development and Validation of a Methylated Marker for Early Diagnosis of Prostate Cancer. Transl. Oncol..

